# Evolution of the Incidence of Oral Cavity Cancers in the Elderly from 1990 to 2018

**DOI:** 10.3390/jcm12031071

**Published:** 2023-01-30

**Authors:** Alice Renou, Anne-Valérie Guizard, Emilien Chabrillac, Gautier Defossez, Pascale Grosclaude, Sophie Deneuve, Sébastien Vergez, Bénédicte Lapotre-Ledoux, Sandrine D Plouvier, Agnès Dupret-Bories, FRANCIM Network

**Affiliations:** 1 Department of Surgery, University Cancer Institute Toulouse—Oncopole, Hôpitaux Universitaires de Toulouse, 31009 Toulouse, France; 2 French Network of Cancer Registries, 31073 Toulouse, France; 3 General Tumor Registry of Calvados, Centre François Baclesse, 14000 Caen, France; 4 ANTICIPE U 1086 Inserm-UCN, 14000 Caen, France; 5 Department of Surgery, Institut Claudius Regaud, University Cancer Institute Toulouse—Oncopole, 31009 Toulouse, France; 6 General Cancer Registry of Poitou-Charentes, Pôle Biologie, Pharmacie et Santé Publique, CHU/Université de Poitiers, 86000 Poitiers, France; 7 Tarn Cancer Registry, Claudius Regaud Institute, University Cancer Institute Toulouse—Oncopole, 31009 Toulouse, France; 8 CERPOP, UMR 1295 Inserm Toulouse III University, 31000 Toulouse, France; 9 Department of ENT, Rouen University Hospital, 76000 Rouen, France; 10 Quantification en Imagerie Fonctionnelle-Laboratoire d’Informatique, du Traitement de l’Information et des Systèmes Equipe d’Accueil 4108 (QuantIF-LITIS EA4108), University of Rouen, 76000 Rouen, France; 11 Somme Cancer Registry, CHU Amiens, CEDEX 1, 80054 Amiens, France; 12 CHIMERE, Surgery, Imaging and Tissue REgeneration of the Cephalic Extremity-Morphological and Functional Characterization, 7516 UR UPJV, CHU-Amiens Picardie, 1 Rond Point du Professeur Cabrol, 80000 Amiens, France; 13 General Cancer Registry of Lille Area, GCS-C2RC, 59000 Lille, France

**Keywords:** elderly, epidemiology, incidence, oral cavity cancer

## Abstract

Objectives: To describe the evolution of the incidence of oral cavity cancers (OCC) among elderly patients in France between 1990 and 2018 and to compare it to the incidence of other cancers sharing the same main risk factors. Material and Methods: The incidence of cancers in mainland France from 1990 to 2018 was estimated from incidence data observed in every cancer registry of the Francim network. Incidence was modeled by a 2-dimensional penalized spline of age and year of diagnosis, associated with a random effect corresponding to the registry. The elderly population was divided into two groups: 70–79 years old and ≥80 years old. Results: There was a 72% increase in the number of OCC cases in women over 70 years of age between the periods 1990–1999 and 2010–2018. As for men, there was a stabilization in the number of cases (+2%). Over the same period, for laryngeal and hypopharyngeal cancers, there was a decrease in incidence in elderly men and an increase in elderly women, although less marked than for OCC. Conclusions: Since the 1990s, the incidence of OCC has been increasing in elderly subjects in France, particularly in women. Population aging and growth or alcohol and tobacco consumption alone do not seem to explain this increase, which is not observed in the same proportions for other upper aerodigestive tract cancer subsites sharing the same main risk factors.

## 1. Introduction

Oral cavity cancers (OCC) represent more than 25% of head and neck cancers in France, coming in second place after oropharyngeal cancers [1]. In nearly 95% of cases, the histological type is squamous cell carcinoma [2].

They represent a public health issue for multiple reasons. Firstly, they have a poor prognosis, with a 5-year standardized survival rate of 49%, mainly due to their late diagnosis at advanced stages [3]. Secondly, OCC patients often have significant comorbidities and an increased risk of developing second primary cancers [4]. Finally, the standard treatment for OCC includes surgery in most cases, which may involve the resection of anatomical structures that are essential for speech, swallowing, or breathing [5]. This management can, therefore, heavily impact the patient’s quality of life [5].

The scientific literature describes a first peak of incidence around the age of 60 years and a second peak around the age of 80 years [1]. The main risk factors (RF) for OCC are alcohol and tobacco consumption [6]. This is also true for laryngeal, hypopharyngeal and oropharyngeal cancers in France, despite the increasing proportion of human papillomavirus (HPV)-related oropharyngeal cancers [6].

In order to reduce the consequences of tobacco and alcohol consumption as well as the incidence of squamous cell carcinoma of the upper aerodigestive tract (UADT), several prevention campaigns have been successfully carried out in France and throughout the world [7]. Consequently, men’s tobacco smoking decreased significantly until the 2000’s. On the other hand, women’s tobacco smoking increased progressively until the mid-1980’s, when it stabilized [8]. Since the 1990’s, men’s alcohol consumption has been steadily decreasing, and that of women has stabilized [9].

Despite these efforts, the worldwide incidence of OCC in the elderly has not decreased and even seems to be on the rise [10]. An increased incidence of this cancer among women aged over 70 years has been described in Western countries, with limited association with smoking and alcohol abuse [11]. This evolution cannot be entirely explained by the aging of the population and the evolution of alcohol and tobacco consumption. The implication of repeated denture traumas or toxic agents has also been suspected [12,13,14]. The presence and evolution of a late incidence peak in France have not been studied to date.

The main objective of this study is to describe the evolution of the incidence of OCC among elderly subjects in France between 1990 and 2018 and to compare it to those of other cancers sharing the same main RF, i.e., laryngeal and hypopharyngeal cancers.

## 2. Materials and Methods

### 2.1. Study Design

This is a descriptive epidemiological study using estimates of cancer incidence in mainland France. These estimates have been obtained by the analysis of incidence data from the 19 regions covered by a general cancer registry, i.e., 22% of the mainland population and approximately 14 million people [1].

We included all patients aged over 15 years newly diagnosed with OCC, laryngeal, or hypopharyngeal cancer between 1990 and 2018, regardless of histological type. Hematological malignancies were not included.

According to the International Classification of Diseases in Oncology (https://icd.who.int/ (accessed on 1 May 2019)), OCC includes the following groups: cancers of the mobile tongue (C02), gum (C03), the floor of the mouth (C04), palate (C05), vestibule, inner cheek and retromolar trigone area (C06). Laryngeal cancers are classified as C32 and hypopharyngeal as C12 and C13.

This study did not require any ethics committee approval as only anonymous, and public data were used.

### 2.2. Statistical Analysis

We considered subjects aged 70 years or more as elderly. This threshold is clinically relevant and regularly used in scientific literature, i.e., the common age limit for the use of chemotherapy in head and neck cancer patients [15]. Elderly subjects were then divided into 2 groups: 70–79 years and 80 years or more.

The incidence in mainland France from 1990 to 2018 was estimated with the incidence data from all general cancer registries of the Francim network. The methodology used has been detailed and validated in a previous study [16]. In summary, the national incidence was estimated using registries’ incidence data only, without using mortality as a correlate of the incidence, since the area covered by the registries was considered representative of mainland France in terms of cancer incidence. It was derived from a Poisson model in which the incidence rate was modeled by a two-dimensional penalized spline of age and year of diagnosis plus a random effect corresponding to the registry. This two-dimensional model was compared to simpler models (without age-year interaction or year effect), using the Akaike information criterion to select the best model in terms of parameter adequacy and parsimony. The numbers presented herein were obtained by this modeling.

The presented incidence rates are age-specific incidence rates calculated for the following periods: 1990–1999, 2000–2009, and 2010–2018. Results for 2016–2018 are projections based on the incidence data from up to 2015.

The population data used (population, population by region, sex, year and annual age between 1990–2018) were made available by the French National Institute of Statistics and Economic Studies (INSEE).

## 3. Results

### 3.1. Number of Cases

Among women aged 70 years or more, between the 1990–1999 and 2010–2018 periods, there was an increase of 72% in the cases of OCC (3093–5323 cases). Over the same time interval, we noted an increase of 17.2% (887–1040 cases) and 60% (312–499 cases) in laryngeal and hypopharyngeal cancer cases, respectively (Table 1).

Over the 2010–2018 period, women aged 80 years or more accounted for 24.7% of new OCC cases (12.3% and 7.3% for larynx and hypopharynx, respectively), compared with 19.9% across the 1990–1999 period (10.6% and 6.1% for larynx and hypopharynx, respectively) (Table 2).

Among men aged 70 years or more, between the 1990–1999 and 2010–2018 periods, we observed the stabilization of the number of OCC cases (6734–6851 cases; +1.7%). Over the same time interval, we noted a decrease of 17% (9392–7820 cases) and 21% (5559–4376 cases) in laryngeal and hypopharyngeal cancer cases, respectively (Table 1).

Over the 2010–2018 period, males aged 80 years or more accounted for 9.2% of new OCC cases (9.6% and 7.2% for larynx and hypopharynx, respectively), compared with 3.8% across the 1990–1999 period (5.9% and 3.3% for larynx and hypopharynx, respectively) (Table 2).

### 3.2. Incidence Rates 

**Figure 1:**
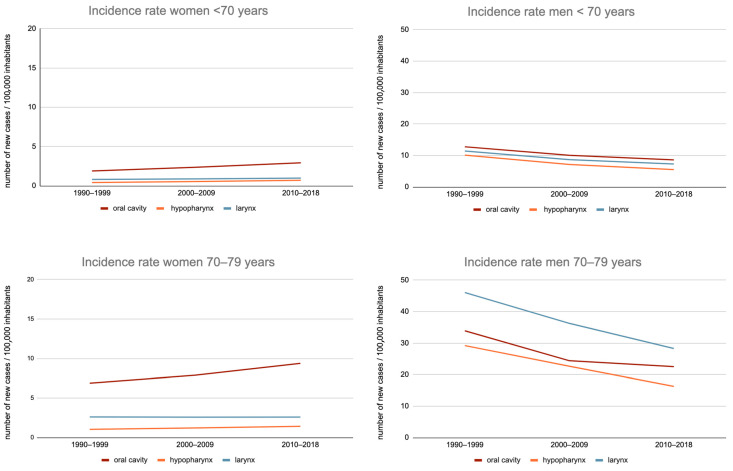

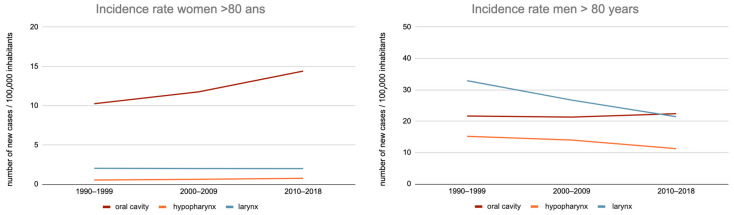
Evolution of the incidence rate of the oral cavity, hypopharynx, and larynx cancer in France, between 1990–1999 and 2010–2018, by sex, among subjects aged less than 70 years, 70 to 79 years, and 80 years or more.

Among women, between the 1990–1999 and 2010–2018 periods, there was a 36.3%-increase in the OCC incidence rate (specific rate increased from 6.9 to 9.4/100,000) in subjects aged 70–79 years and a 40.5%-increase (10.2 to 14.4/100,000) in those aged 80 years or more (Figure 1).

This trend was also observed for hypopharyngeal cancers: incidence rates increased from 36.9% (from 1 to 1.4/100,000) in the 70–79 age group and 40% (from 0.5 to 0.8/100,000) in those aged 80 or more.

On the other hand, the incidence of laryngeal cancer remained stable over this period for these two populations: 2.6–2.6/100,000 and 2.0–2.0/100,000.

Among men, between the 1990–1999 and 2010–2018 periods, there was a 33.5%-decrease in OCC incidence rates in the 70–79 age group (33.9–22.5/100,000) and an increase of 3.4% (21.7–22.4/100,000) in the 80 years or more age group.

Nevertheless, for the 70–79 years age group, the decrease in incidence between the 2000–2009 and 2010–2018 periods was only 8.4% (24.4–22.5/100,000), thus with an overall trend towards stabilization.

Regarding hypopharyngeal and laryngeal cancers, there was a greater decrease in incidence rates in the age groups “70–79 years” (−44.3% and −38.6%, respectively) and “80 or more” (−25.8% and −34.9%, respectively).

**Figure 2:**
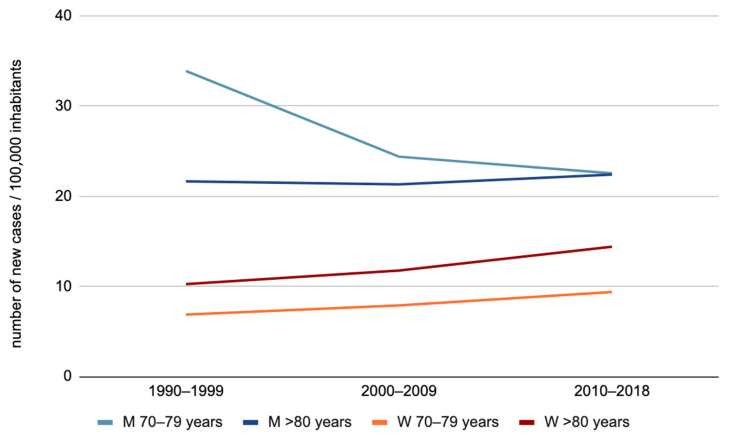
Evolution of oral cavity cancer incidence by sex for the “70–79 years” and “80 years or more” age groups (M: men, W: women).

In summary (Figure 2), we observed an increase in the OCC incidence among women aged 70–79 years and 80 years or more, as well as among men aged 80 years or more. Regarding men aged 70–79 years, an overall trend toward the stabilization of OCC incidence was observed.

## 4. Discussion

### 4.1. Incidence of Oral Cavity Cancers Worldwide

Between 2012 and 2018, OCC has receded from being the sixth to the eighteenth most incident cancer worldwide [17]. Overall, its incidence and mortality are greater in men than in women. South East Asia (India, Sri Lanka) and the Pacific Islands have the highest incidence and mortality rates because of high exposure to the main RF, including betel quid chewing [18]. Incidence rates were highest in Papua New Guinea, i.e., 27.5 and 15.1 per 100,000 person-years in males and females, respectively [18].

Since the 1990s, the age-standardized incidence rate has been showing a decreasing trend in men and an increasing trend in women, especially in European countries and in some developed countries, such as Japan. This increase often mirrors the evolution of alcohol and tobacco consumption in these countries [18,19]. In France, over the same period, there was a 122% increase in the number of OCC cases in women regardless of age, of which 29% were attributable to population increase, 26% to its aging and 67% to an actual increase of the risk of OCC [1].

Recent literature regarding this matter has mainly focused on the incidence surge among young subjects (<45 years) worldwide, but also in France, and seems to imply the involvement of unknown RF [20,21]. However, American and European studies also suggested a trend for a rising incidence among elderly subjects, with a limited association with smoking and alcohol abuse [11,22,23].

On the other hand, the epidemiology of this cancer among elderly subjects has poorly been studied in the literature. A Danish study showed an increase in OCC incidence in men and a stabilization among women for all age groups from 1980 to 2012. For both sexes, incidence rates were the highest in the population aged 70 years or more [23]. The Dutch national incidence data (https://iknl.nl/nkr-cijfers (accessed on 1 October 2021)) also show an increase for both sexes (age groups 70–74, 75–79, 80–84, and >85 years). Notably, smoking habits among men decreased more slowly in these countries than in France over that period [24]. Thus, we could assume that the sharp decrease in exposure to the major RF in elderly men in France could hide an increase in OCC cases among non-smoking and non-drinking patients (decreasing incidence in elderly men in France, increasing incidence among elderly women in France and among both sexes in neighboring countries).

**Figure 3:**
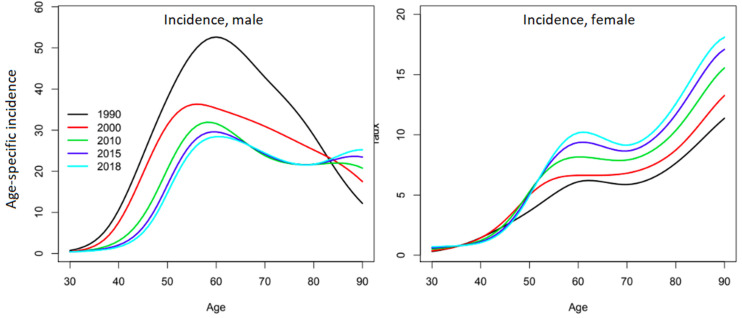
Evolution of age-specific incidence of oral cavity cancers for French men and women. Reprinted with permission from Defossez, G. et al. [1] (p. 39) Copyright 2019, Francim_HCL_SPF_INCa. Available online: https://www.santepubliquefrance.fr/maladies-et-traumatismes/cancers/cancer-du-sein/documents/rapport-synthese/estimations-nationales-de-l-incidence-et-de-la-mortalite-par-cancer-en-france-metropolitaine-entre-1990-et-2018-volume-1-tumeurs-solides-etud (accessed on 5 July 2019).

The official report from which this study’s data were extracted presents the changes in the age-specific incidence curves over time and shows very different evolutions for each sex (Figure 3) [1]. Among men, the major incidence peak around 60 years of age decreased until 2010, when a second peak seemed to appear after 80 years of age. Among women, the overall incidence is significantly lower. In the 1990s, the incidence rate increased with age until it reached a plateau between 60 and 70 years of age, then further increased after 70 years of age and reached a maximum at the highest ages. Since then, the incidence has been steadily increasing for all ages, but more rapidly around the age of 60 years. The different trends in the age-specific incidence curves are such that the profiles become increasingly similar between men and women, which supports the hypothesis of the homogenization of the RF among the two sexes.

During the study period, the growth of the French population (from 57.9 million to 66.9 million) and the evolution of the average lifespan (from 72.7 years to 79.6 years for men and from 81 years to 85.5 years for women) during this study undeniably contributes to the evolution of the number of cancer cases. However, these evolutions are, to a large extent, taken into account in our analysis, notably because we present specific rates by age group and because this study is based on a comparison of the incidences of the different cancers studied in the same population. Regardless of these evolutions, this study shows an increase in the incidence of OCC among elderly subjects in France since 1990, namely an increase in women aged 70 years or more and in men aged 80 years or more, with a slowdown in the decline in incidence among men aged 70 to 79 years over the 2000–2009 period. Furthermore, according to the comparison of the incidences of the different cancers studied in the same population, such an increase has not been observed for men in other locations or in women for laryngeal cancers. The incidence rise seems comparable to hypopharynx cancer in women, but the low incidence rates and the number of cases do not allow us to draw conclusions. To our knowledge, this increase in the incidence of hypopharyngeal cancers in elderly women has not been described to date.

### 4.2. Risk Factors

The implication of known RF does not seem to fully explain the evolution of OCC incidence. As a matter of fact, the reduction of smoking and alcohol abuse in men since the 1950s should be mirrored by a comparable decrease in the incidence of OCC, even among men aged 80 years and over [8]. In 2015, the ICARE (Investigation of occupational and environmental CAuses of REspiratory cancers) study group estimated that nearly 20% of OCC in France were not related to alcohol and/or tobacco exposure [25]. The prevalence of oral HPV-16 infections is increasing, yet some studies suggest that it would be involved in the oncogenesis of only 2 to 3% of OCC [26]. The hypothesis of an additional RF should therefore be investigated [8,9].

Other possible etiologies are nutritional RF, such as red meat consumption, or a body-mass index ≤ 18.5 [27,28]. Conversely, the regular consumption of fruits, vegetables, tea, olive oil, folate, and natural vitamin C appears to have a protective effect [29,30]. Another study from the ICARE group has reported a dose-response relationship between OCC occurrence in women and exposure to trichloroethylene (a solvent used in the rubber, cleaning and painting industries) [13].

Poor dental hygiene also appears to be at risk, although it is hard to isolate it from its associated RF (tobacco, alcohol, low socioeconomic status, and low fruit and vegetable consumption) [14,31]. The chronic use of alcohol-based mouthwash is another potential RF for this cancer [32]. Oral microbiome alterations could also play a role in head and neck carcinogenesis [33]. Fungal oral infections, and Candida albicans in particular, could enable the development of oral dysplasia and squamous cell carcinoma [34].

An increasing number of authors are focusing on the association between denture-wearing and OCC development. According to a 2014 meta-analysis, the risk of OCC is significantly greater with wearing dentures (OR 1.42) and particularly with ill-fitting dentures (OR 3.90) [12]. Among patients without usual RF, cancer occurrence is more frequent in certain subsites, such as lateral tongue, gingiva, and floor of the mouth, which may be related to teeth or prostheses trauma [35]. Two hypotheses have been raised to explain this: repeated mucosal microtrauma leading to carcinogenesis through an inflammation process or chronic mechanical stress [36].

### 4.3. Strengths and Limitations of the Study

This study benefits from an exhaustive data collection of the new cases of cancer, allowing true-to-life national estimates. Few articles have reported the evolution of OCC epidemiology in the international community, and this is, to our knowledge, the first study focusing on elderly patients [10].

However, this data collection method also has weaknesses. On the one hand, extrapolation into national estimates may be less accurate among population groups where the incidence is low, such as laryngeal and hypopharyngeal cancers in women. On the other hand, data from registries do not provide information about exposure to RF or tumor characteristics. Finally, the evolution of the incidence curves for patients aged 80 years or more may be influenced by an improvement in the diagnosis and management of OCC since the 1990s. This hypothesis cannot be entirely ruled out. It would result in improved survival due to earlier diagnosis or better treatment [37]. Indeed, over the same period, the 1- and 5-year net survival at the age of 70 and 80 years has improved (respectively +6% and +9% at 1 year, +10% and +12% at 5 years). However, this improvement is comparable to the improvement observed for the younger age groups [3].

It can also be mentioned that cancers of the soft palate were categorized into “oral cavity,” whereas they are generally considered oropharyngeal cancers in clinical practice. As HPV-related oropharyngeal cancers primarily arise from lymphoid structures (not the soft palate), our findings are likely not biased by the surge in HPV-related oropharyngeal cancers. Oropharyngeal cancer cases were not studied here because of their specific oncogenesis and evolving epidemiology during this period, making their epidemiology not comparable to that of OCC.

This study does not provide information about tumor stages or affected oral cavity subsites. Of note, Dutch studies based on their national registry suggested an increased incidence for stages I and IV as well as for the mobile tongue and gum subsites [38]. This is consistent with other studies reporting that mobile tongue and gum cancers were less frequently associated with alcohol and tobacco consumption than other subsites [25,35].

### 4.4. Impact on Clinical Practice

The management of elderly patients with OCC seems to be more and more frequent in head and neck surgeons’ practice. This study confirms an increase in the number of cases and quantifies it. Indeed, OCC in the elderly is nowadays a rising healthcare burden, and the number of new cases in the 80 years or more age group has almost doubled between the 1990–1999 and 2010–2018 periods (3099 and 5746, respectively). The survival improvement in this population will also lead to rising healthcare needs, as it will increase the number of prevalent cases to follow up.

It seems important that every physician involved in OCC management (ENT specialists, dentists, oral and maxillofacial surgeons, prosthetists, and general practitioners) is aware of this increased incidence among elderly women who are free of alcohol and tobacco exposure. The objective is to promote early diagnosis and access to curative surgical treatments for better survival and quality of life [37].

## 5. Conclusions

Since the 1990s, the incidence of OCC has been increasing among elderly subjects, especially women, in several countries, according to recent data from the literature. Our study confirms this notion through the example of France. Aging, population growth, and the evolution of alcohol and tobacco consumption seem insufficient to explain this surge since it has not been observed in the same proportions for other head and neck cancer sites sharing the same main RF.

These observations highlight the importance of geriatric management in the daily practice of head and neck oncology. They also raise the need for studies regarding the preferentially involved subsites and potential unknown RF, such as mucosal contact agents (denture wearing, alcohol-based mouthwashes, etc.), poor dental hygiene, and nutritional risk factors.

## Figures and Tables

**Table 1:** Evolution of the number of cases of oral cavity, hypopharynx, and larynx cancers by sex, among patients aged 70 years or more between 1990 and 2018.

Site	Period	Women ≥ 70 Years		Men ≥ 70 Years	
		Number of Cases	Evolution of the Number of Cases between 1990 and 2018 (%)	Number of Cases	Evolution of the Number of Cases between 1990 and 2018 (%)
Oral cavity	1990–1999	3093	+72	6734	+2
	2000–2009	4302		6747	
	2010–2018	5323		6851	
Hypopharynx	1990–1999	312	+60	5559	−21
	2000–2009	444		5743	
	2010–2018	499		4376	
Larynx	1990–1999	887	+17	9392	−17
	2000–2009	1067		9584	
	2010–2018	1040		7820	

**Table 2:** The proportion of patients with oral cavity, hypopharynx, and larynx cancers by age and sex category, for the 1990–1999, 2000–2009, and 2010–2018 periods.

Site	Period	Women				Men			
		<70 Years *n* (%)	70–79 Years *n* (%)	≥80 Years *n* (%)	Total	<70 years *n* (%)	70–79 Years *n* (%)	≥80 Years *n* (%)	Total
Oral cavity	1990–1999	4919 (61.4)	1501 (18.7)	1592 (19.9)	8012	32,972 (8)	5227 (13.2)	1507 (3.8)	39,706
	2000–2009	6361 (59.65)	2122 (19.9)	2180 (20.4)	10,663	26,781 (79.9)	4823 (14.4)	1924 (5.7)	33,528
	2010–2018	7390 (58.13)	2186 (17.2)	3137 (24.7)	12,713	21,457 (75.8)	4242 (15)	2609 (9.2)	28,308
Hypo pharynx	1990–1999	1080 (77.6)	227 (16.3)	85 (6.1)	1392	26,133 (82.5)	4502 (14.2)	1057 (3.3)	31,692
	2000–2009	1465 (76.7)	326 (17.1)	118 (6.2)	1909	19,073 (76.9)	4479 (18)	1264 (5.1)	24,816
	2010–2018	1783 (78.1)	332 (14.6)	167 (7.3)	2282	13,770 (75.9)	3063 (16.9)	1313 (7.2)	18,146
Larynx	1990–1999	2114 (70.4)	570 (19)	317 (10.6)	3001	29,499 (75.8)	7101 (18.3)	2291 (5.9)	38,891
	2000–2009	2382 (69.1)	695 (20.1)	372 (10.8)	3449	23,139 (70.7)	7172 (21.9)	2412 (7.4)	32,723
	2010–2018	2482 (70.5)	605 (17.2%)	435 (12.3)	3522	18,174 (69.9)	5322 (20.5)	2498 (9.6)	25,994

Percentages are the ratio of patients in each age group to all subjects of the same sex affected by a cancer of the same site over the same period.
